# Dengue at the time of COVID-19 in the Philippines

**DOI:** 10.5365/wpsar.2020.11.2.015

**Published:** 2021-05-07

**Authors:** Xerxes T. Seposo

**Affiliations:** Correspondence to Xerxes Seposo (seposo.xerxestesoro@nagasaki-u.ac.jp).

Cases of infection with severe acute respiratory syndrome coronavirus 2 (SARS-CoV-2), the virus responsible for coronavirus disease 2019 (COVID-19), have been increasing since the virus emerged in Wuhan, China, in December 2019. As of 13 March 2021, confirmed COVID-19 cases have exceeded 119 million infected individuals across 188 countries, with more than 2.6 million recorded deaths. (*1*) National health systems have attempted to contain the pandemic through control measures such as community quarantine and isolation. In the Philippines, an enhanced community quarantine (ECQ) took effect on 15 March 2020 in an effort to flatten the epidemic curve. (*2*) ECQ involves placing stringent limitations on people’s mobility and strict regulations on various industry operations, all of which are enforced by uniformed personnel. (*3*) In spite of the ECQ, active infections have been steadily increasing in the country, at 611 618 total cases and 12 694 deaths as of 13 March 2021. (*1*)

In 2020, the Philippines recorded a substantial decrease in the number of dengue cases, with a reduction in notified cases of about 70–90% during the rainy season (*4*) specifically from epidemiological weeks 28 to 40. (*5*) Apart from existing control and prevention measures implemented in the country – such as the establishment of dengue centres of excellence in tertiary hospitals and the creation of dengue fast lanes – the decrease in the number of cases during the COVID-19 pandemic may be largely due to the reduced mobility of the population. Several studies noted that reduction of localized household movement could lead to a reduction in transmission. (*6*) On a larger geographical scale, movement control measures reportedly slow or even prevent the spread of a dengue epidemic from locations with high transmission intensity to suburbs or remote areas. (*7*) Conversely, the decrease may have also been a result of reporting hesitancy due to the fear of contracting COVID-19 while visiting a health facility. In Caribbean and Latin American countries, an initial sharp decrease in dengue cases coincided with the start of reporting of COVID-19 cases. (*8*) The reduction in dengue trend may be due, in part, to the impact of the pandemic on health-seeking behaviour of the population, driven by fear of being infected. A similar reduction in health facility visits was also purported to be the reason behind the decrease in both infectious diseases and non-infectious diseases during the pandemic. (*9*) The Philippines has experienced several clusters of infection in hospitals. COVID-19 hospital transmissions have been widely documented in hospitals in various countries. (*3*) The existence of these hospital clusters has decreased medical-seeking behaviour due to the fear of contracting the disease, to the extent that it has impacted the reporting of other diseases and illnesses.

Several other countries in the World Health Organization (WHO) Western Pacific Region also noted a decrease in dengue cases in 2020. (*5*) However, this was not the case in Singapore, which has seen a substantial increase in cases, possibly associated with the country’s physical distancing measures implemented in response to COVID-19. (*10*) For example, the work-from-home measure implemented may have contributed to the increase in dengue cases, compared with the usual workplace setting. Compared with workplaces, residences have a higher propensity for causing dengue infection, owing to the thriving conditions for mosquito breeding. The rise in dengue cases in Singapore and the reduction in the Philippines and other countries in the region show how different control measures (e.g. mobility restrictions) can vary in their effects on levels of dengue. These variations may be due to the extent and degree of control measures, coupled with prevention and control measures directed to either dengue or COVID-19, and inherent country-specific sociodemographic factors; thus, further investigation of these factors is warranted, subject to the availability of data.

The Philippines and other countries in the WHO Western Pacific Region did not see a similar increase in dengue cases in 2020. However, caution should be exercised, because a trend of increasing dengue cases could still develop in current conditions. The renewed rise of COVID-19 cases and the roll-out of COVID-19 vaccinations may have an impact on dengue cases in the latter part of 2021. The increase in COVID-19 cases may lead to more stringent control measures, but the strength of these measures will depend on the progress of vaccination coverage. According to the Philippines’ current COVID-19 vaccination timeline, the general population will probably start receiving vaccinations in July 2021, after completion of the full master list of people to be vaccinated, which is expected by 30 June 2021. (*1*) The dengue season starts a month later, at the end of July.

In summary, although the Philippines has seen a decrease in dengue cases in 2020, a scenario in which cases increase is possible, as has happened in Singapore. Further investigation of countries in the region is needed to ascertain which factors have affected the varying impact on notified dengue cases from COVID-19-related measures, compounded by innate sociodemographic characteristics. Nevertheless, health managers can plan ahead and appraise the current conditions, including the rise in COVID-19 cases and vaccination progress, and consider how these may affect the number of dengue cases in the latter part of 2021.
